# Pelvic abscess due to *Mycoplasma hominis* following caesarean section

**DOI:** 10.1099/jmmcr.0.005059

**Published:** 2016-08-30

**Authors:** Nobuaki Mori, Aya Takigawa, Narito Kagawa, Tsuyoshi Kenri, Shinji Yoshida, Keigo Shibayama, Yasuko Aoki

**Affiliations:** ^1^​ Department of General Internal Medicine, National Hospital Organization Tokyo Medical Center, 2-5-1 Higashigaoka, Meguro-ku, Tokyo 152-8902, Japan; ^2^​ Department of Gynecology Medicine, National Hospital Organization Tokyo Medical Center, 2-5-1 Higashigaoka, Meguro-ku, Tokyo 152-8902, Japan; ^3^​ Department of Clinical Laboratory, National Hospital Organization Tokyo Medical Center, 2-5-1 Higashigaoka, Meguro-ku, Tokyo 152-8902, Japan; ^4^​ Department of Bacteriology II, National Institute of Infectious Diseases, 4-7-1 Gakuen, Musashimurayama-shi, Tokyo 208-0011, Japan

**Keywords:** Mycoplasma hominis, pelvic abscess, chorioamnionitis, fever, lower abdominal pain, clindamycin

## Abstract

**Introduction:**

* Mycoplasma hominis* is associated with genito-urinary tract infection and adverse pregnancy outcomes. However, whether the species is a true pathogen or part of the genito-urinary tracts natural flora remains unclear.

**Case presentation:**

A 41-year-old pregnant woman was admitted to our hospital at 38 weeks and 5 days of gestation owing to premature rupture of the membranes. The patient delivered by caesarean section. Subsequently, the patient complained of lower abdominal pain and had persistent fever. Enhanced computed tomography revealed pelvic abscesses. Gram staining of pus from the abscess and vaginal secretions indicated presence of polymorphonuclear leucocytes but no pathogens. Cultures on blood agar showed growth of pinpoint-sized colonies in an anaerobic environment within 48 h. Although administration of carbapenem and metronidazole was ineffective and we could not fully drain the abscess, administration of clindamycin led to clinical improvement. The isolates 16S rRNA gene and *yidC* gene sequences exhibited identity with those of *M. hominis.*

**Conclusion:**

Physicians should consider* M. hominis* in cases of pelvic abscesses where Gram staining yields negative results, small colonies are isolated from the abscess and treatment with β-lactam antibiotics is ineffective.

## Introduction

*Mycoplasma hominis*, most frequently isolated from the human genito-urinary tract, is associated with syndromes such as cervicitis and pelvic inflammatory disease and has been linked to adverse pregnancy outcomes (e.g. chorioamnionitis, preterm labor and neonatal infection). Rates of *M. hominis* colonisation vary from 25 % to 67 % among healthy women (Capoccia *et al.*, 2013). However, whether the species is a true pathogen or part of the genito-urinary tracts natural flora remains unclear.

Here, we report a case of pelvic abscesses caused by* M. hominis* in a patient who underwent caesarean section.

## Case report

**Fig. 1.**
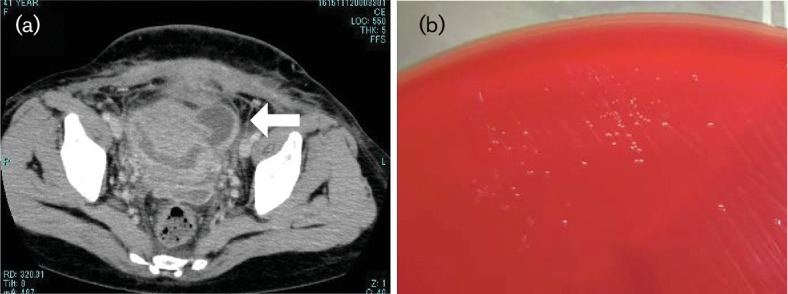
(a) Enhanced computed tomography image showing an abscess around the endocervix (white right arrow). (b) Culture characteristics of *M. hominis.* Pinpoint, translucent colonies are visible on blood agar.

A 41-year-old pregnant woman (gravida 0, para 0) was admitted to our hospital at 38 weeks and 5 days of gestation owing to premature rupture of the membranes. The patient had no significant medical history and was not taking medication. On the admission day, she complained of fever. Laboratory testing indicated leucocytosis (29 800 µl^−1^) and an elevated C-reactive protein level (2.0 mg dl^−1^). The clinical diagnosis was chorioamnionitis. Ampicillin (2 g) was administered intravenously and the patient delivered by caesarean section. The amniotic fluid was uncontaminated. Although cefmetazole was administered (1 g every 8 h) following the operation and the wound appeared clean, the patient complained of lower abdominal pain and had persistent fever. At post-operative day 7, the patient continued to experience symptoms and laboratory tests indicated leucocytosis (19 400 µl^-1^) and elevated C-reactive protein level (13.0 mg dl^−1^). Two sets of blood culture were performed and antibiotic therapy was switched to doripenem (1 g every 8 h). Enhanced computed tomography revealed the formation of an abscess around the endocervix, right paracolic sulcus, under abdominal wall at the left lower abdomen and in the Douglas pouch (Fig. 1a). The patient underwent percutaneous abscess drainage under ultrasound echo guidance. However, we could not drain the abscess fully through the indwelling catheter, and therefore, it was removed after 3 days. Pus from the abscess and vaginal secretions was cultured. Gram staining of both indicated presence of polymorphonuclear leucocytes but no pathogens. However, cultures on blood agar showed growth of pinpoint–sized colonies in an anaerobic environment within 48 h (Fig. 1b). The decision was taken to administer metronidazole (500 mg every 8 h) in addition to the current therapy. However, no changes were observed in the patients symptoms. The isolate could not be identified using the MicroScan Walkaway system (Siemens Healthcare Diagnostics). We performed antimicrobial susceptibility testing and evaluated the minimum inhibitory concentration (MIC) of various antibiotic agents using Dry Plate Eiken (Eiken Chemical) and Brucella Broth (Wako Pure Chemical Industries) for 48 h in an anaerobic environment. The MICs of the antibiotic agents are shown in Table 1. In accordance with these results, antibiotic therapy was switched to a combination of clindamycin (600 mg every 8 h) and ceftriaxone (2 g every 24 h). The patient recovered immediately and continued this treatment for 10 days. The neonate was not infected.

**Table 1.** Antimicrobial susceptibilities for the patient's isolates

Antibiotics	MIC (μg ml^−1^)
Penicillin G	>1
Ampicillin	>1
Cefmetazole	>32
Ceftriaxone	>8
Aztreonam	>16
Meropeneme	>8
Amoxicillin/clavulanate	>8
Piperacillin/tazobactam	>64
Clindamycin	≤0.12
Minocycline	≤0.25
Clarithromycin	>64
Levofloxavcin	0.5

To identify the organism, polymerase chain reaction (PCR) amplification and sequencing were performed to analyse a 16S rRNA gene and a *yidC* gene that was related to a membrane protein of *M. hominis* as previously described (Sasaki *et al.*, 1997; Férandon *et al.*, 2014). We performed sequence analysis (1384 bp) using a GenBank BLAST search. The isolates 16S rRNA gene sequence exhibited identity with *M. hominis* AF1 (GenBank accession number: CP009677.1) and the *yidC* gene exhibited greatest similarity to *M. hominis* AF1 (99 %).

## Discussion

The clinical course observed in this patient suggests that *M. hominis* can cause pelvic abscesses. Physicians should consider *M. hominis* as a causative pathogen of pelvic abscess where treatment with β-lactam antibiotics has been ineffective, in addition to performing Gram staining and culture under aerobic condition where an infection is unidentifiable.

We considered *M. hominis* as the cause of pelvic abscesses because it was the sole organism isolated from the abscess. Carbapenem and metronidazole antibiotics have been shown to be effective in treating pelvic abscess caused by organisms such as *Enterobacteriaceae.* In this case, these were ineffective, and drainage of the pelvis could not be fully performed. However, the patient recovered immediately following administration of clindamycin. In this case, it appears that chorioamnionitis and pelvic abscesses were caused by *M. hominis* in the genito-urinary tract because the organism was isolated from vaginal secretions and the caesarean section wound was clean. We reviewed the previously published cases of pelvic abscess formation due to *M. hominis* following caesarean section, and the results are summarised in Table 2 (Barberá *et al.*, 1995; Yamaguchi *et al.*, 2009; Koshiba *et al.*, 2010; Muin *et al.*, 2015).

**Table 2.** Clinical characteristics of patients with pelvic abscess formation due to *M. hominis* following caesarean section

No.	Age, y	Gravida/para	Underlying disease	Symptoms	Wound infection	Other isolates from abscess	Drainage/ operation	Initial antibiotics	Antibiotics for *M. hominis* infection	Duration of antibiotics for *M. hominis* infection	Neonatal infection	Outcome	Reference
1	38	No description	No description	Fever, hypovolemic	−	None	+	CTX, MNZ	CTX, MNZ, CLDM	21 days	No description	Recover	Barbera J, *et al.*
2	27	2/2	No description	Fever	+	None	+	Cephalosporin, carbapenem, aminoglycoside,CLDM	PZFX	7 days	No description	Recover	Yamaguchi M, *et al.*
3	27	0/0	No description	Fever, erythema	+	None	+	FMOX, IPM/CS	CPFX	14 days	No description	Recover	Koshiba H, *et al.*
4	24	1/0	None	Fever, abdominal pain	−	*Gardnerella vagina*, *Ureaplasma urealyticum*, *Actinobaculum schaalii*	+	AMPC/CVA	AMPC/CVA, CLDM	24 days	No description	Recover	Muin DA, *et al.*
5	41	0/0	None	Fever, abdominal pain	−	None	−	MEPM, MNZ	CLDM, CTRX	10 days	None	Recover	Our case

Abbreviations: CTX, cefotaxim; MNZ, metronidazole; CLDM, clindamycin; FMOX, fulomoxicef; IMP/CS, imipenem/cilastatin; AMPC/CVA, amoxicillin/clavulnic acid; MEPM, meropenem; DOXY,doxycycline; PZFX, pazufloxacin; CPFX, ciprofloxacin; CTRX, ceftriaxion.

With administration of β-lactam antibiotics,* M. hominis* was solely isolated from the abscess in 4 of the 5 cases. In the fourth case, multiple pathogens were isolated, including genito-urinary pathogens such as *U. urealyticum*, for which β-lactam antibiotics are ineffective. Some reports have suggested abscess formation in extragenital regions, such as brain, scalp, perinephric and parapharyngeal abscesses (Pailhoriès *et al.*, 2014; Abdel-Haq *et al.*, 2002; Camara *et al.*, 2007; Kennedy *et al.*, 2009), although this is relatively rare. In these reports, *M. hominis* was also isolated solely from abscesses, but β-lactam antibiotics had been administered in most cases. Physicians should suspect *M. hominis* as a causative agent of abscess when β-lactam antibiotics are ineffective.

Indications for treatment, optimal choice of antibiotics and average duration of *M. hominis* infection remain unknown. β-lactam antibiotics were administered in all cases before the causative pathogen was identified as* M. hominis*. However, as *M. hominis* lacks peptidoglycan, it is naturally resistant to β-lactam antibiotics. Clindamycin and fluoroquinolones have been the antibiotics of choice in all cases. Due to undesirability of administering tetracycline and fluoroquinolones during pregnancy and lactation, clindamycin is an acceptable agent. Therefore, we suggest that clindamycin is the most appropriate empiric therapy while awaiting culture results, especially if no improvement is observed. All cases except ours describe treatment of the abscess caused by* M. hominis* using a combination of surgical drainage or operation and systemic antibiotics. In our case, clindamycin administration led to clinical improvement, although we could not fully drain the abscess. We considered that clindamycin was so effective because it is characterised by penetrating well into abscesses and is actively taken up and concentrated by phagocytes and polymorphonuclear leucocytes; moreover, the MIC for the *M. hominis* isolate in our case was low. Additionally, improvement was observed over a relatively short duration of antibiotic therapy.

Identification of *M. hominis* is often challenging, and infection is often underdiagnosed as it is a slow-growing bacterium without a cell wall. *M. hominis* may grow and produce small colonies on standard media but can be cultured more successfully on designed media. However, molecular-based techniques using PCR and 16S rDNA sequencing may be essential for identifying *M. hominis* (Taylor-Robinson & Lamont, 2010). Matrix-assisted laser desorption/ionisation time-of-flight mass spectrometry has also been shown to be effective (Pailhoriès *et al.*, 2014). Physicians and microbiologists should suspect *M. hominis* based on clinical history, Gram staining and colony morphology. Where feasible, the use of molecular methods could assist diagnosis.

Thus, *M. hominis* can cause pelvic abscess. Physicians should suspect* M. hominis* when Gram staining yields negative results, small colonies are isolated from the pelvic abscess and treatment with β-lactam antibiotics is ineffective.
